# Sensitivity improvement of quartz-enhanced photoacoustic spectroscopy using the stochastic resonance method

**DOI:** 10.1016/j.pacs.2025.100707

**Published:** 2025-02-27

**Authors:** Yingchao Xie, Hao Xiong, Shiling Feng, Ning Pan, Chuan Li, Yixuan Liu, Ye Zhang, Ligang Shao, Gaopeng Lu, Kun Liu, Guishi Wang

**Affiliations:** a School of Earth and Space Sciences, University of Science and Technology of China, Hefei, Anhui 230026, China; b College of Environmental Science and Optoelectronic Technology, University of Science and Technology of China, Hefei, Anhui 230026, China; c Laboratory of Atmospheric Physico-Chemistry, Anhui Institute of Optics and Fine Mechanics, Chinese Academy of Sciences, Hefei, Anhui 230000, China

**Keywords:** Quartz-enhanced photoacoustic spectroscopy (QEPAS), Stochastic resonance (SR), Trace gas sensor, Signal enhancement

## Abstract

Quartz-enhanced photoacoustic spectroscopy (QEPAS) is a promising technique for trace gas sensing, offering advantages such as compact size and high sensitivity. However, noise remains a critical factor limiting detection sensitivity. In this study, a novel approach was proposed to leverage noise for the enhancement of weak QEPAS signals. The method employs stochastic resonance (SR), which counterintuitively utilizes noise to amplify weak spectral signals, thereby significantly improving the signal-to-noise ratio of the QEPAS sensor. The effectiveness of this approach was demonstrated through methane (CH₄) detection using QEPAS. Experimental results indicate that the SR algorithm enhances the output signal by a factor of 3 and reduces the minimum detection limit (MDL) from 329 ppb to 85 ppb compared to conventional QEPAS. The proposed SR-enhanced algorithm presents a promising strategy for further improving QEPAS sensor performance in trace gas detection.

## 1 Introduction

Methane (CH_4_) is a potent trace greenhouse gas with a significantly higher global warming potential than carbon dioxide (CO₂) but a comparatively shorter atmospheric lifetime of approximately ten years. This relatively short persistence implies that effective CH₄ emission control can substantially mitigate the rate of global warming. The Global Methane Commitment (GMC) represents an important step in this direction, necessitating precise monitoring of CH₄ emissions for the development of effective environmental policies and regulations. However, achieving high-precision CH₄ detection remains challenging [1], [2], [3], [4], [5], [6], particularly in applications such as early-stage natural gas leak detection, regional emission measurements, and quantitative assessments, all of which impose stringent requirements on sensor sensitivity. Improving the capability of trace gas sensing systems to extract weak characteristic signals is essential for addressing these challenges.

Various spectroscopic techniques have been extensively employed for trace gas detection in atmospheric monitoring [7], [8], [9], [10], [11], [12], [13], [14], [15], [16], [17]. Among them, quartz-enhanced photoacoustic spectroscopy (QEPAS) enables highly sensitive, background-free detection by converting laser absorption of the target gas into acoustic and thermal energy using a cost-effective quartz tuning fork (QTF). Despite the strong potential of QEPAS-based sensors for trace gas monitoring, system noise remains a major limitation, adversely affecting measurement stability. This challenge is particularly pronounced in the near-infrared (NIR) overtone absorption region, which, while advantageous due to its low-cost and low-power consumption characteristics, exhibits inherently weak absorption. Consequently, achieving high-precision continuous monitoring of atmospheric background CH_4_ remains a significant obstacle. Given that the photoacoustic signal strength increases with higher laser power, erbium-doped fiber amplifiers (EDFAs) have been explored as a means to amplify weak signals [18], [19], [20], [21], [22]. However, EDFA-based amplification degrades beam quality, potentially increasing noise in the QEPAS sensing system [20]. Moreover, the use of fiber amplifiers such as EDFAs or Raman fiber amplifiers (RFAs) to enhance the signal-to-noise ratio (SNR) presents additional challenges, including increased system complexity and reduced miniaturization potential, particularly in specialized bands that require custom fiber amplifiers. Traditional noise suppression algorithms, including the Savitzky-Golay (S-G) filter [23], [24], Kalman filter (KF) [25], [26], and wavelet transform (WT) filter [27], [28], are widely employed due to their low cost, ease of implementation, and effectiveness in improving the SNR without increasing system complexity. However, when the spectral bands of the signal and noise overlap, these filtering techniques inadvertently attenuate the useful signal, leading to waveform distortion and reduced accuracy in concentration measurements.

Unlike conventional noise suppression strategies, stochastic resonance (SR) [29] offers a counterintuitive yet effective approach to weak signal enhancement. By leveraging noise of an optimal intensity, SR can significantly improve the output SNR. While extensively studied across various disciplines [30], [31], [32], [33], [34], its practical application in trace gas sensing remains underexplored.

This study introduces a QEPAS system integrated with an SR algorithm for highly sensitive CH_4_ detection. A 1651-nm NIR distributed feedback (DFB) laser was employed as the excitation source, with the SR algorithm incorporated to enable high-precision measurements of low-concentration CH₄. Unlike conventional filtering techniques that suppress noise at the cost of signal attenuation, the SR algorithm enhances the detection of weak signals embedded in noise, thereby improving sensitivity while maintaining system cost-effectiveness.

## 2 Theory and methodology

In a QEPAS system, the absorption of a modulated laser beam by the target gas induces thermal expansion, which, in turn, causes the QTF to oscillate at a characteristic frequency. The resulting QTF vibration signal, denoted as s, can be expressed as

(1) $$s∼\frac{\alpha \mathrm{PQ}}{f_{0}}$$

where α is the spectral absorption coefficient, *P* represents the laser power, and *Q* denotes the quality factor of the quartz tuning fork. Enhancing QEPAS system performance primarily relies on two approaches: increasing the acoustic signal strength and suppressing noise.

The stochastic resonance (SR)-based QEPAS system leverages specific noise characteristics to amplify weak signal detection. This synergistic interaction between the photoacoustic signal, noise, and the SR system can be described by a first-order nonlinear Langevin equation:

(2) $$\;\frac{\mathrm{dx}}{\mathrm{dt}}=-\frac{\mathrm{dU}\left(x\right)}{\mathrm{dx}}+s\left(t\right)+D\xi (t)$$

where x is the output, $U\left(x\right)$ represents the system potential function, $s\left(t\right)$ and ξ(t) represent the photoacoustic signal and Gaussian white noise varying with time t, respectively, and D denotes the noise intensity.

For non-periodic signals in QEPAS, adopting a monostable SR system simplifies implementation while mitigating waveform distortion in the output signal, a common issue with bistable systems during signal processing [35]. The system potential function *U*(*x*) in a monostable SR system is expressed as

(3) $$U\left(x\right)=a+\frac{1}{4}bx^{4}$$

where a and b are potential function parameters. Substituting Eq. (3) into Eq. (2) results in

(4) $$\;\frac{\mathrm{dx}}{\mathrm{dt}}=-bx^{3}+s(t)+D\xi (t)$$

In the context of QEPAS, this equation can be rewritten as follows:

(5) $$\;\frac{dS_{\mathrm{out}}(t)}{\mathrm{dt}}=\;-b*{S_{\mathrm{out}}}^{3}\left(t\right)+S_{\mathrm{in}}\left(t\right)+D\xi (t)$$

where $S_{\mathrm{in}}\left(t\right)$ represents the 2ƒ signal inputted by the QEPAS system into the SR algorithm, and $S_{\mathrm{out}}(t)$ represents the corresponding photoacoustic spectral signal output. Compared to the Eulerian scheme, the discrete stochastic differential Eq. (5) is more accurately solved using the fourth-order Runge-Kutta method [35].

**Fig. 1:**
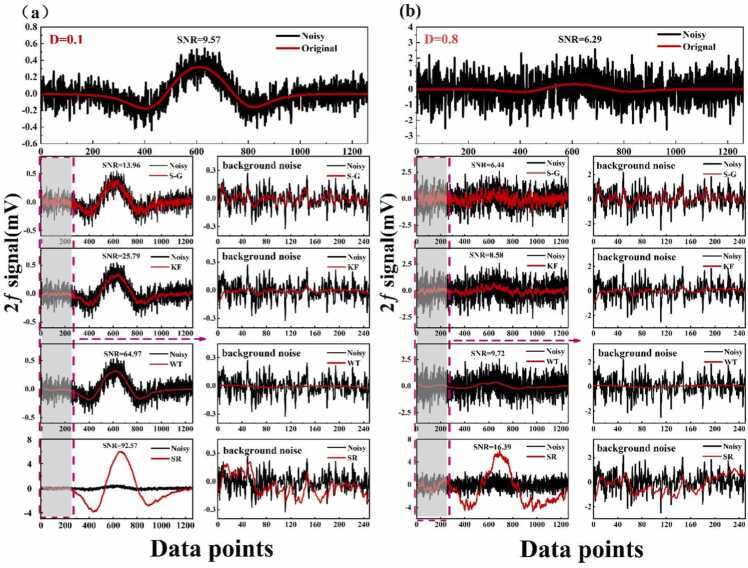
Performance of different algorithms at varying noise intensities: (a) *D* = 0.1. (b) *D* = 0.8.

To evaluate the SR algorithm’s effectiveness in extracting weak signals under varying noise conditions, a 500 ppm CH₄ signal with a high SNR was experimentally obtained and used as a noise-free reference. This assumption is justified, as the added noise significantly exceeds the background noise. Different levels of Gaussian noise were then introduced to simulate spectral signals under various noise conditions, with noise intensity quantified by the standard deviation D. The resulting low-SNR signals were processed using different algorithms, each optimized to maximize SNR while preventing signal distortion. The performance of each algorithm under optimal parameters is illustrated in Fig. 1

The output SNR was used to assess the effectiveness of each algorithm, defined as the ratio of the peak signal to the standard deviation of the baseline. The SR algorithm enhanced the signal peak by approximately 20-fold compared to the original signal peak. Although the SR method led to a slight increase in baseline standard deviation, the overall improvement in useful signal strength outweighed this effect. This trade-off resulted in a superior SNR for spectral signals processed with the SR algorithm compared to conventional filtering techniques. In scenarios with extreme noise levels (*D* = 0.8), noise-suppression-based filtering algorithms attenuated both noise and the useful signal, leading to waveform distortion and compromising gas concentration quantification. In contrast, the SR algorithm effectively extracted spectral signals despite strong noise interference, substantially enhancing the detection of low-concentration gases in practical sensing applications and trace gas monitoring in complex environmental conditions.

## 3 Experimental setup

**Fig. 2:**
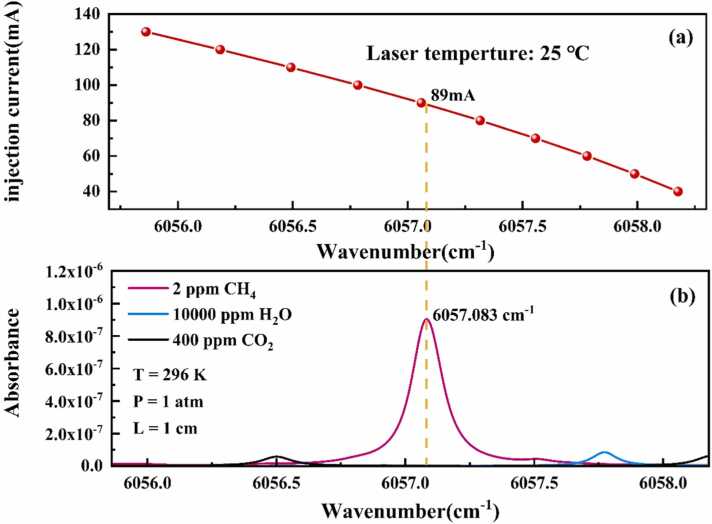
(a) Laser injection current characteristics as a function of emitted wave count at 25°C. (b) Simulated absorption coefficients of CH₄, CO₂, and H₂O using the HITRAN database at 296 K, 1 atm pressure, and an optical path length of 1 cm.

A 1651 nm NIR DFB laser was selected as the light source. Although CH₄ absorption in the NIR region is weaker than in the mid-infrared region, NIR DFB lasers are widely used for high-precision CH₄ sensing due to their low cost and mature fabrication technology. Fig. 2(a) illustrates the laser’s injection current characteristics as a function of the emitted wave count at an operating temperature of 25 °C. The laser's scanning range, controlled by the injection current, spans from 6055.861 cm⁻¹ to 6058.178 cm⁻¹ , fully covering the CH₄ absorption spectrum. Using the HITRAN database, the absorption coefficients of 2 ppm CH₄ were simulated under ambient conditions of 296 K, 1 atm pressure, and an effective optical path length of 1 cm. As shown in Fig. 2(b), the CH₄ absorption peak appears at a wave number of 6057.083 cm⁻¹ , corresponding to a DFB laser drive current of 89 mA. Considering that spectral interference from other gases can notably impact concentration inversion accuracy in the QEPAS system, the absorption spectra of 400 ppm CO_2_ and 1 % H₂O were also simulated under identical conditions. As depicted in Fig. 2(b), the spectral overlap among H₂O, CO₂, and CH₄ is minimal, confirming that interference from these gases is negligible.

**Fig. 3:**
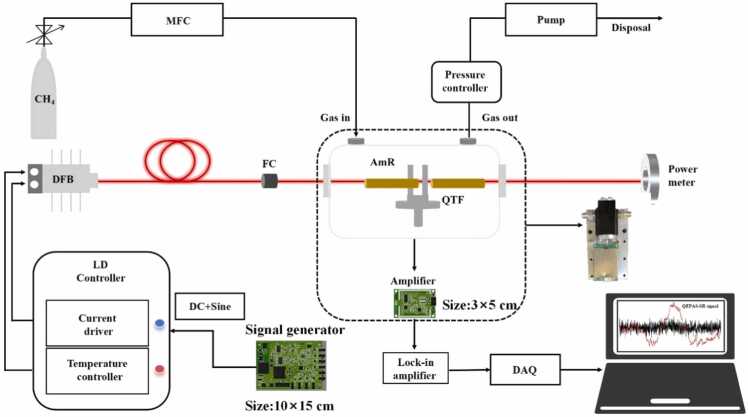
Schematic of the QEPAS system for CH_4_ detection.

**Fig. 4:**
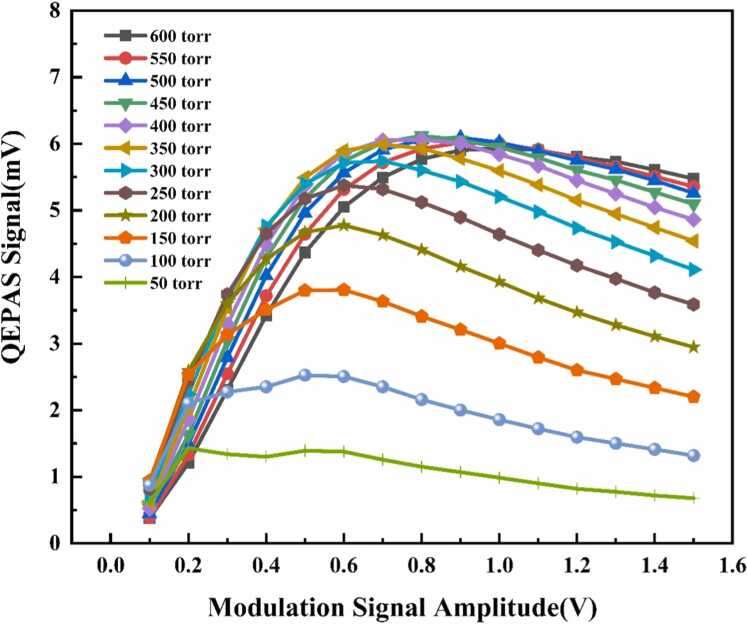
Peak QEPAS signal of 1000 ppm CH₄ under different pressures and modulation amplitudes.

A schematic of the QEPAS sensing system is presented in Fig. 3. The NIR DFB laser is controlled by an integrated current driver and temperature controller. A high-precision temperature controller maintains the laser's operating temperature at 25°C, minimizing wavelength drift caused by temperature fluctuations, which could affect measurement accuracy. Photoacoustic signals are detected using wavelength modulation with second-harmonic detection. The resonance frequency *f*0 the QTF is determined via frequency sweeping correction using a custom-built function generator (10 × 15 cm). This generator produces a sinusoidal modulation signal at *f*0/2, optimizing resonance-enhanced photoacoustic signals from the QTF. The laser scanning range (75–105 mA) is modulated by a DC signal from the function generator, ensuring complete coverage of the CH₄ absorption spectrum. The laser beam is collimated using a fiber-optic collimator before passing through the gas chamber, two acoustic micro-resonators (AmRs), and the QTF, which enhances acoustic signal intensity. An optical power meter is used to precisely align the laser, ensuring optimal incidence. The piezoelectric current signal generated by the QTF undergoes three-stage amplification using a self-developed preamplifier (3 × 5 cm) before being demodulated by a lock-in amplifier. The processed signals are captured by a data acquisition card (DAQ) and transferred to a computer, where a LabVIEW-based stochastic resonance (SR) algorithm is applied to extract weak second-harmonic 2 f signals from background noise. Both modulation amplitude and gas chamber pressure significantly influence the detection performance of the QEPAS system. To determine the optimal conditions, spectral signals were evaluated at various pressure and modulation amplitude combinations using 1000 ppm CH₄ standard gas. A flow controller regulated the inlet flow rate of CH₄, while a high-precision pressure controller (accuracy: ± 0.01 torr) maintained the gas chamber pressure. Fig. 4 presents the peak 2ƒ signals of 1000 ppm CH₄ under different pressure and modulation amplitude conditions. The maximum QEPAS signal was observed at a pressure of 450 torr and a modulation amplitude of 0.8 V. Therefore, these values were selected as the system's optimal operating conditions.

## 4 Results and analysis

### 4.1 SR in the QEPAS system

**Fig. 5:**
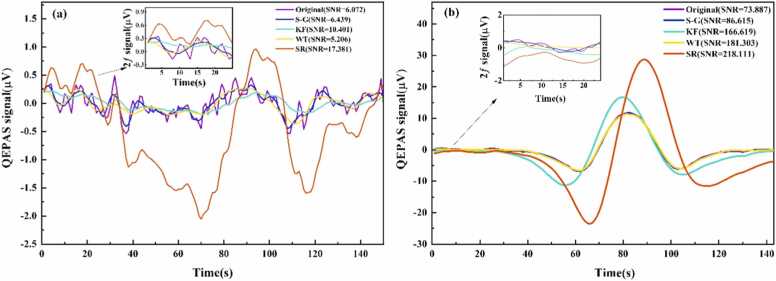
Comparison of the SR algorithm with other filtering algorithms for photoacoustic signals at different CH₄ concentrations: (a) 2 ppm and (b) 50 ppm.

The performance of the SR algorithm in real-world trace gas sensing applications was evaluated using a QEPAS system with CH₄ standard gas at two concentrations (2 ppm and 50 ppm) under optimal modulation amplitude and pressure conditions. The denoising effects of different algorithms at these concentrations are shown in Fig. 5(a) and (b).

**Fig. 6:**
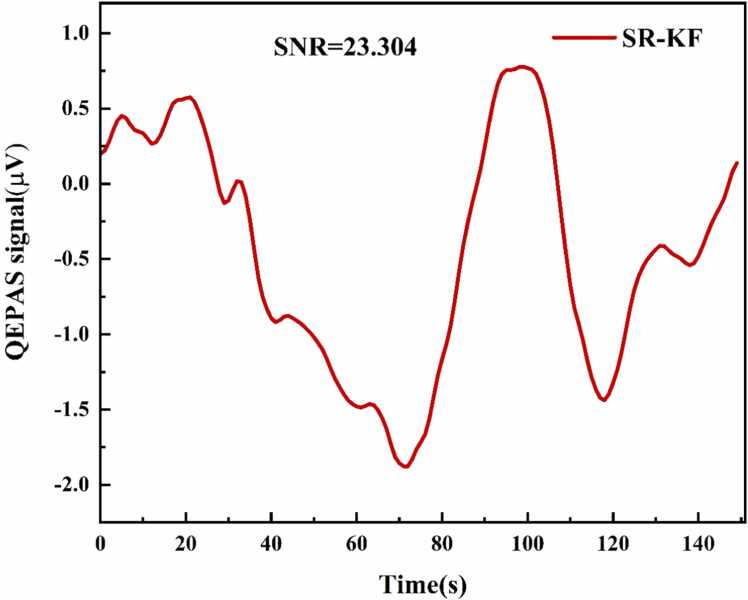
Effect of the SR algorithm combined with the KF algorithm on the 2 ppm CH₄ photoacoustic signal.

As shown in Fig. 5(a), detecting weak 2 ppm CH_4_ signals in real-world applications presents a greater challenge than processing simulated Gaussian noise added to the 2 f signal. Although the WT algorithm exhibits superior performance with simulated data, its output SNR is considerably lower in practical scenarios. This is primarily due to the trade-off between noise suppression and signal distortion, which often affects noise suppression-based filtering algorithms when applied to weak spectral signals at low concentrations. The SR algorithm demonstrates superior performance in this context. Despite a slight increase in baseline standard deviation (approximately 1.0 times its original value), the peak signal is enhanced by a factor of 2.9, while the output SNR increases from 6.1 to 17.3. Consequently, the minimum detection limit (MDL) is reduced from 329 ppb to 115 ppb. As shown in Fig. 5(b), the WT algorithm performs well for the 50 ppm CH₄ signal, achieving a high SNR, in contrast to its weaker performance at low concentrations. Given the effectiveness of filtering algorithms for high-SNR signals and the SR algorithm’s tendency to slightly amplify baseline standard deviation, a secondary filtering step was applied to further improve detection performance. The Kalman Filtering (KF) algorithm was selected for this purpose. As shown in Fig. 6, combining the SR and KF algorithms enhances the output SNR by a factor of 3.8 compared to the original signal, reducing the detection limit to 85 ppb.

### 4.2 Performance analysis of SR-based QEPAS system

**Fig. 7:**
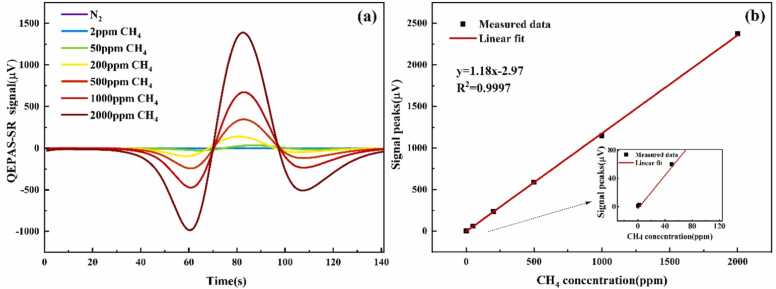
(a) 2 *f* signals from the SR-based QEPAS system at various CH₄ concentrations. (b) Peak 2 *f* signal as a function of CH₄ concentration.

To validate the linear response of the SR-based QEPAS system (QEPAS-SR), the gas chamber was filled with CH₄ standard gas at various concentrations (2, 50, 200, 500, 1000, and 2000 ppm), as well as pure nitrogen for reference. Fig. 7(a) presents the 2 f signals corresponding to different CH₄ concentrations. As depicted in Fig. 7(b), a strong linear correlation was observed between the SR output and CH₄ concentration, with a coefficient of determination (R²) of 0.9997.

**Fig. 8:**
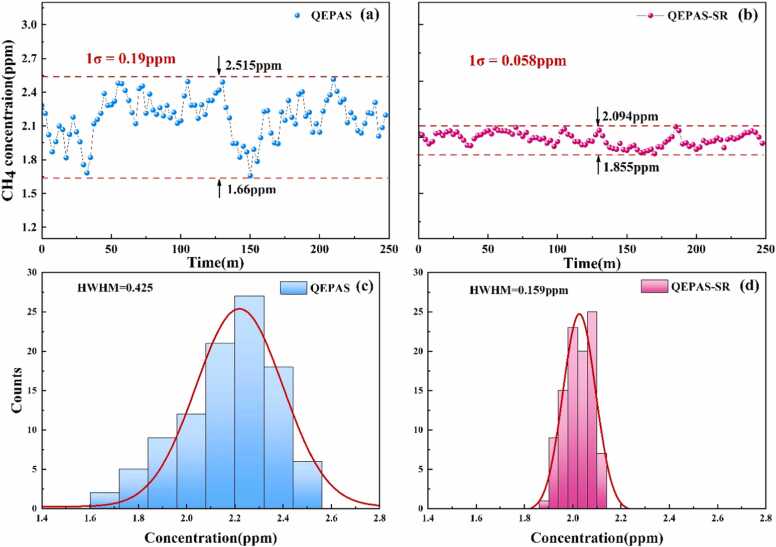
Continuous CH₄ concentration measurements over 4 h: (a) QEPAS and (b) QEPAS-SR. Statistical histograms of (c) QEPAS and (d) QEPAS-SR.

The long-term stability of the system was also evaluated. The gas chamber was filled with 2 ppm CH₄ standard gas, and continuous measurements were conducted for approximately 4 h under controlled laboratory conditions. The measurement results for both the standard QEPAS system and the SR-enhanced QEPAS-SR system are shown in Fig. 8(a) and (b). The QEPAS-SR system outperformed the standard QEPAS system by a factor of 3.3, significantly improving detection sensitivity from 190 ppb to 58 ppb. Statistical histograms of the QEPAS and QEPAS-SR measurements, presented in Fig. 8(c) and (d), exhibit Gaussian distributions with half-width at half-maximum (HWHM) values of 425 ppb and 159 ppb, respectively. Additionally, Table 1 compares the performance of the QEPAS-SR sensor with advanced QEPAS sensors reported in previous studies. Highly sensitive CH₄ sensors in the NIR band, such as those proposed in [37], incorporate an additional RFA to enhance detection; however, this increases system overhead and poses challenges for miniaturization and integration. By contrast, the software-based SR algorithm can be seamlessly integrated with other high-sensitivity sensors, further improving weak signal detection without increasing system complexity or cost, owing to the advantages of software-based approaches, such as portability and ease of implementation.

**Table 1:** Comparison of CH_4_ detection limits in QEPAS methods.

Reference	Laser	Wavenumber (cm^−1^)	MDL (ppb) (@1 s)
[36]	NIR-DFB	6057.08	711
[37]	NIR-DFB+RFA	6057.08	47
[38]	NIR-DFB	6046.95	1241
[39]	NIR-DFB	6057.08	3200
This work	NIR-DFB	6057.08	85

## 5 Conclusions

A high-sensitivity CH₄ QEPAS sensor was developed, with the integration of a software-based SR algorithm significantly enhancing detection performance without increasing system complexity. The effectiveness of the QEPAS-SR system was systematically evaluated using experimental data collected in a controlled laboratory environment. Results indicate that the SR algorithm improves the SNR of the QEPAS system by a factor of 2.86 while reducing the MDL from 329 ppb to 115 ppb. Additionally, in practical sensing applications, the SR algorithm effectively amplifies signal peaks, leading to a substantial improvement in SNR, though it also introduces a slight increase in baseline standard deviation. To mitigate this effect, the KF algorithm was applied for secondary filtering, further reducing the MDL to 85 ppb. Continuous measurements conducted over a four-hour period demonstrated a 3.28-fold enhancement in detection sensitivity compared to the conventional QEPAS system. These findings confirm that the SR algorithm significantly improves weak signal detection in real-world trace gas sensing applications. By leveraging noise to enhance the output SNR, the SR algorithm exhibits strong potential for trace gas detection in high-noise environments. Further performance enhancements may be achieved through the use of custom QTFs.

## CRediT authorship contribution statement

**Wang Guishi:** Writing – review & editing. **Liu Kun:** Supervision, Project administration, Funding acquisition. **Pan Ning:** Formal analysis. **Feng Shiling:** Software. **Xiong Hao:** Writing – original draft, Software. **Xie Yingchao:** Writing – review & editing, Writing – original draft. **Shao Ligang:** Software. **Zhang Ye:** Supervision. **Liu Yixuan:** Data curation. **Li Chuan:** Supervision. **Lu Gaopeng:** Supervision.

## Declaration of Competing Interest

The authors declare that they have no known competing financial interests or personal relationships that could have appeared to influence the work reported in this paper.

## Data Availability

Data will be made available on request.
