# Supplementary material for: Sensitivity improvement of quartz-enhanced photoacoustic spectroscopy using the stochastic resonance method
Source: Photoacoustics. 2025 Feb 27;43:100707. doi: 10.1016/j.pacs.2025.100707 (PMC11919392; doi:10.1016/j.pacs.2025.100707)
Supplement: Supplementary file 1 — Supplementary material [file mmc1.docx]

**Supporting Information**

**Sensitivity improvement of quartz-enhanced photoacoustic spectroscopy using the stochastic resonance method**

Yingchao Xie^a,1^, Hao Xiong^b,c,1^, Shiling Feng^c^, Ning Pan^c^, Chuan Li^c^, Yixuan Liu^c^, Ye Zhang^c^, Ligang Shao^c^, Gaopeng Lu^a^, Kun Liu^c,*^,Guishi Wang^c,**^

^a^School of Earth and Space Sciences, University of Science and Technology of China, Hefei, Anhui 230000, China

^b^College of Environmental Science and Optoelectronic Technology, University of Science and Technology of China, Hefei, Anhui 230026, China

^c^Laboratory of Atmospheric Physico-Chemistry, Anhui Institute of Optics and Fine Mechanics, Chinese Academy of Sciences, Hefei 230000, Anhui, China

*Corresponding author. Kun Liu, Laboratory of Atmospheric Physico-Chemistry, Anhui Institute of Optics and Fine Mechanics, Chinese Academy of Sciences, Hefei, China.

**Corresponding author. Guishi Wang, Laboratory of Atmospheric Physico-Chemistry, Anhui Institute of Optics and Fine Mechanics, Chinese Academy of Sciences, Hefei, China.

*[liukun@aiofm.ac.cn](mailto:liukun@aiofm.ac.cn)

[**gswang@aiofm.ac.cn](mailto:**gswang@aiofm.ac.cn)

^1^The two authors contribute equally to this work and should be considered co-first authors.

**Table of Contents**

**S1**. Parameter selection for the SR algorithm and other filtering algorithms

**S2.** Solving stochastic resonance output signals by the fourth-order Runge-Kutta method

**S3**. SR algorithm processing flow

**S1. Parameter selection for the SR algorithm and other filtering algorithms**

For the SR algorithm, a smaller parameter b provides a higher signal-to-noise ratio, but also introduces signal distortion. The parameter b was set to 1.81 × 10^-7^ while ensuring that the signal is not distorted.For the Kalman filtering algorithm, the settings of the process noise covariance (Q) and the measurement noise covariance (R) have a direct impact on the calculation of the Kalman gain, and thus on the degree of confidence the filtering algorithm places in the predicted and measured values. After weighing the signal-to-noise ratio and hysteresis of the signal and the loss of signal quality, Q and R were set to 100, and 0.2, respectively.The key parameters of the S-G algorithm include the window width and the polynomial order, which were set to 11 and 3, respectively, in this experiment.In this experiment, the WT algorithm chose to use a soft-threshold denoising with a wavelet decomposition of 6 layers.

**S2. Solving stochastic resonance (SR) output signals by the fourth-order Runge-Kutta method**

In a quartz-enhanced photoacoustic spectroscopy (QEAPS) system, the output signal of the SR can be obtained by solving the discrete equations:

$\frac{dS_{out}(t)}{dt}= -b*{S_{out}}^{3}\left( t \right)+S_{in}\left( t \right)+D\xi(t)$ (1)

where $S_{in}\left( t \right)$ and $S_{out}(t)$ are the SR input and output signals, respectively, that vary with time t. b is an adjustable parameter, and $D$ and $\xi(t)$ are the noise intensity as well as Gaussian noise, respectively. Equation (1) can be solved by the fourth-order Runge-Kutta method. The method can be described as follows：

$$S_{out}\left( n \right)=S_{out}\left( n-1 \right)+\frac{1}{6}h*\left( k_{1}+2k_{2}+2k_{3}+k_{4} \right), n=1,2,3\ldots.N$$

$k_{1}=-b\left( S_{out}\left( n-1 \right) \right)^{3}+S_{in}\left( n-1 \right)$

$k_{2}=-b\left( S_{out}\left( n-1 \right)+\frac{k_{1}}{2} \right)^{3}+S_{in}\left( n-1 \right)$

$k_{3}=-b\left( S_{out}\left( n-1 \right)+\frac{k_{2}}{2} \right)^{3}+S_{in}\left( n \right)$

$k_{4}=-b\left( S_{out}\left( n-1 \right)+k_{3} \right)^{3}+S_{in}\left( n \right)$

where $S_{in}\left( n \right)$ and $S_{out}\left( n \right)$ are the discrete inputs and outputs, respectively, and N is the input length. h is the reciprocal of the sampling frequency.

**S3. SR algorithm processing flow**

The SR algorithm is suitable for nonlinear systems. To improve the signal-to-noise ratio using the SR algorithm in a QEPAS system, it is first necessary to normalize the signal to ensure that all inputs have the same intensity. The SR algorithm processing flow is as follows:

Step1. Acquisition of photoacoustic signals and normalization of their amplitude.

Step2. Initialize parameter b, input the acquired signal and solve the output signal by fourth-order Runge-Kutta method.

Step3. Adjust parameter b to select the optimal parameter b while ensuring that the signal is not distorted.

Step4. The SR output signal is obtained under the optimal parameters. The final QEPAS-SR output is obtained by inverse normalization of the SR output signal.
